# Hypofractionated Radiotherapy for Palliation of Main Portal Vein Tumor Thrombosis

**DOI:** 10.3389/fonc.2022.882272

**Published:** 2022-04-27

**Authors:** Fang Fang, Bin Qiu, Peng Zhen, Junjie Wang

**Affiliations:** ^1^ Department of Radiation Oncology, Chifeng Tumor Hospital, Chifeng, China; ^2^ Department of Radiation Oncology, Peking University Third Hospital, Beijing, China

**Keywords:** hypofractionated radiotherapy, portal vein tumor thrombosis, hepatocellular carcinoma, cirrhosis, palliation

## Abstract

**Background:**

Hypofractionated radiotherapy delivered for portal vein tumor thrombosis (PVTT) located in the main portal vein is rarely exploited. The study aimed to evaluate the efficacy and safety of hypofractionated radiotherapy as palliative treatment for PVTT in cirrhotic patients with hepatocellular carcinoma.

**Methods:**

From March 2016 to July 2020, 16 patients (mean age, 59.1 ± 6.3 years; 15 men) with hepatocellular carcinoma and hepatitis virus-related cirrhosis who underwent hypofractionated radiotherapy for PVTT (located in the main portal vein) in our institute were retrospectively reviewed.

**Results:**

Complete response of the PVTT was observed in 4 cases (25%) with partial response in 7 cases (43.75%) and stable disease in 5 cases (31.25%). Symptom relief was observed in all 7 patients suffering from ventosity. The median time to progression was 6 months (interquartile range, IQR: 6–12 months). Eight patients (50%) failed due to primary cancer progression, 7 patients failed due to extrahepatic metastasis, and only 1 patient failed due to PVTT progression. The median overall survival was 17.4 months (IQR: 8–25 months). Grade I/II anorexia/nausea was observed in 14 patients (87.5%) and Grade I/II leukopenia was observed in 14 patients (87.5%). No complications ≥ Grade III were observed.

**Conclusions:**

Hypofractionated radiotherapy as palliative treatment appears effective and safe for PVTT located in the main portal vein in cirrhotic patients with advanced hepatocellular carcinoma, yielding a high rate of tumor response. Further study is warranted.

## Background

Hepatocellular carcinoma (HCC) is the third leading cause of cancer-related death worldwide. Advanced HCC is usually the case when the cancer is initially diagnosed due to the asymptomatic nature of HCC. Portal vein tumor thrombosis (PVTT) is frequent in patients with advanced HCC and has been reported in as many as 44%–84% of patients from autopsy data and in 31%–50% from clinical data (1). The prognosis for these patients remains discouraging with a median survival of only 2.7 months without treatment (2).

The recommended therapeutic methods for PVTT are systemic therapy usually with targeted drugs or recently reported atezolizumab plus bevacizumab regimen (3–8), which is promising while unlikely to be a cost-effective option (9). Notably, PVTT can obstruct portal venous flow and worsen portal hypertension (10). An approach using local therapy and systemic agents before progression should be investigated (11). In selected patients with good hepatic reserves, surgical resection and radioembolization may be attempted (11–13). However, these local therapies were either invasive or inexhaustive and only applicable to a small range of patients.

Recently, external beam radiation therapy (EBRT) was recommended as a non-invasive local therapeutic option for PVTT, and promising results have been noted (12–17). However, hypofractionated radiotherapy for PVTT located in the main portal vein is rarely exploited (18). Hypofractionated radiotherapy was conducted as a palliative treatment for PVTT of the main portal vein in our institute. Here, the study aimed to evaluate the efficacy and safety of hypofractionated radiotherapy as palliative treatment for PVTT located in the main portal vein only in cirrhotic patients with hepatocellular carcinoma.

## Methods

### Study Design

The retrospective study complies with the Declaration of Helsinki and was approved by our institutional review board. The written informed consent was obtained. The documented clinical data from March 2016 to July 2020 in our institute were retrospectively reviewed. Cirrhotic patients with advanced hepatocellular carcinoma who underwent hypofractionated radiotherapy for the main PVTT only were included. The indication of hypofractionated radiotherapy was the intent to control PVTT in the main portal vein in cirrhotic patients with or without portal hypertension symptoms. The contraindications of hypofractionated radiotherapy were as follows: (i) uncontrolled intrahepatic/extrahepatic lesion; (ii) liver function of Child–Pugh Class C; (iii) renal failure; (iv) active portal hypertension, confirmed by upper endoscopy and the presence of symptoms (i.e., gastrointestinal bleeding or refractory ascites); (v) poor condition with Eastern Cooperative Oncology Group (ECOG) score > 2 or expected life span < 1 month; and (vi) pregnancy. All patients had signed an informed consent form for hypofractionated radiotherapy using Gamma-knife.

### Definitions

The PVTT response, symptom relief, time to progression (TTP), overall survival, and complication after hypofractionated radiotherapy were recorded. The PVTT response was evaluated based on the consensus of 2 investigators according to the Response Evaluation Criteria in Solid Tumors on the first computed tomography (CT) or magnetic resonance imaging (MRI) image within 3 months after hypofractionated radiotherapy (19). Symptom relief was recorded according to the patient’s complaint at the first-time follow-up after hypofractionated radiotherapy. TTP was defined as the time from the hypofractionated radiotherapy initialization until tumor progression including not only PVTT but also any intrahepatic or extrahepatic tumor progression. Overall survival was defined as the interval between hypofractionated radiotherapy initialization and death from any cause. Complications were determined by the Common Terminology Criteria for Adverse Events (CTCAE) v4.0 (CTCA) (20).

### Study Population

A total of 16 patients (mean age, 59.1 ± 6.3 years; 15 men) were included in the analysis. All the patients were clinically diagnosed with Barcelona Clinical Liver Cancer (BCLC) stage C HCC according to the European Society for Medical Oncology (ESMO) Clinical Practice Guidelines (21), along with hepatitis B (*n* = 15)/C (*n* = 1) virus-related cirrhosis. The PVTTs were all in the main portal vein (Vp4) and diagnosed by both enhanced CT and MRI according to the Japan criterion (22). Fourteen (87.5%) patients had portal hypertension, while only 1 (6.25%) patient had ascites and 7 (43.8%) patients had ventosity. The liver function of the patients was Child–Pugh stage A in 12 patients and stage B in 4 patients, all with ECOG performance score 1. The summary of the patients is listed in **Table 1**. Ten (62.5%) patients combined with previous or post-treatment for primary tumor control. Only one patient (No. 16) received Lenvatinib and another patient (No. 13) received anti-PD-1; the remaining patients refused systemic therapy due to medical charges. Two patients (Nos. 2 and 14) could not receive transarterial chemoembolization (TACE) before radiotherapy because of PVTT and portal vein recanalization after radiotherapy provides TACE treatment opportunity.

**Table 1:** Summary of the 16 patients.

Patients	Sex	Age	CP	PH	Combined treatment	Dose/fraction	Symptom relief	PVTT-R	Failure	TTP	OS	Complications	Prognosis
1	M	55	B	Yes	Pre-TACE (PD)	45 Gy/15F	Ventosity relief; Ascites reduced	SD	Primary cancer progression	3	6.9	None	Died due to primary tumor progression
2	F	66	A	Yes	Post-TACE	8.8 Gy/4F+26.4 Gy/11F	Ventosity relief	PR	Extrahepatic metastasis	24	31.3	Grade 2 anorexia/nausea	Died due to extrahepatic metastasis
3	M	55	B	Yes	None	45 Gy/15F	Ventosity relief	CR	Extrahepatic metastasis	6	20.5	Grade 2 anorexia/nausea; Grade 1 leukopenia	Died due to extrahepatic metastasis
4	M	47	A	Yes	None	36.4 Gy/13F	Ventosity relief	PR	Primary cancer progression	12	25.0	Grade 1 anorexia/nausea; Grade 1 leukopenia	Died due to cachexia
5	M	58	A	Yes	Pre-TACE (PD)	33.8 Gy/13F	Without Symptom	PR	Extrahepatic metastasis	12	17.6	Grade 2 anorexia/nausea; Grade 2 leukopenia	Died due to cachexia
6	M	63	A	Yes	Post-HIFU for primary	25 Gy/10F	Ventosity relief	SD	Primary cancer progression	3	7.7	Grade 2 anorexia/nausea; Grade 1 leukopenia	Died due to hepatorenal syndrome and extrahepatic metastasis
7	M	56	A	Yes	Pre-TACE+ RT (PR); Post-TACE	45 Gy/15F	Without symptom	CR	Primary cancer progression	12	44.2	Grade 1 anorexia/nausea; Grade 1 leukopenia	Died due to tumor progression
8	M	64	A	Yes	None	39 Gy/13F	Without symptom	CR	Extrahepatic metastasis	12	17.4	Grade 2 anorexia/nausea; Grade 2 leukopenia	Died due to liver failure and extrahepatic metastasis
9	M	58	A	Yes	None	45 Gy/15F	Ventosity relief	PR	Extrahepatic metastasis	12	16.4	Grade 2 anorexia/nausea; Grade 2 leukopenia	Died due to liver failure and extrahepatic metastasis
10	M	58	A	Yes	Pre-HAIC	39 Gy/13F	Without symptom	PR	Extrahepatic metastasis	12	14.6	Grade 1 anorexia/nausea; Grade 2 leukopenia	Died due to cachexia and extrahepatic metastasis
11	M	55	A	Yes	Pre-+post-TACE	45 Gy/15F	Without symptom	CR	Primary cancer progression	36	40.5	Grade 2 anorexia/nausea; Grade 2 leukopenia	Alive
12	M	51	A	Yes	None	39 Gy/13F	Without symptom	PR	PVTT progression	3	12	Grade 2 anorexia/nausea; Grade 2 leukopenia	Alive
13	M	68	A	Yes	Post-anti-PD-1	45 Gy/15F	Ventosity relief	SD	Primary cancer progression	6	8.6	Grade 1 anorexia/nausea; Grade 1 leukopenia	Died due to variceal bleeding
14	M	71	B	Yes	Pre-RFA (CR); Post-TACE	45 Gy/15f	Without symptom	PR	Primary cancer progression	6	10.2	Grade 1 anorexia/nausea; Grade 1 leukopenia	Alive
15	M	62	A	No	None	30 Gy/10F	Without symptom	SD	Primary cancer progression	6	6	Grade 2 anorexia/nausea; Grade 1 leukopenia	Died due to tumor progression
16	M	58	B	No	Pre-TACE+ Lenvatinib	45 Gy/15f	Without symptom	SD	Extrahepatic metastasis	6	6.4	Grade 1 leukopenia	Alive

CP, Child–Pugh; PH, portal hypertension; M, male; F, female; Pre-, previous; Post-, post-Gamma-knife radiotherapy; PVTT-R, portal vein tumor thrombosis response; TACE, transarterial chemoembolization; HIFU, high-intensity focused ultrasound; RT, radiotherapy; PD, progression disease; PR, partial response; CR, complete response; TTP, time to progression; OS, overall survival.

### Hypofractionated Radiotherapy

**Figure 1:**
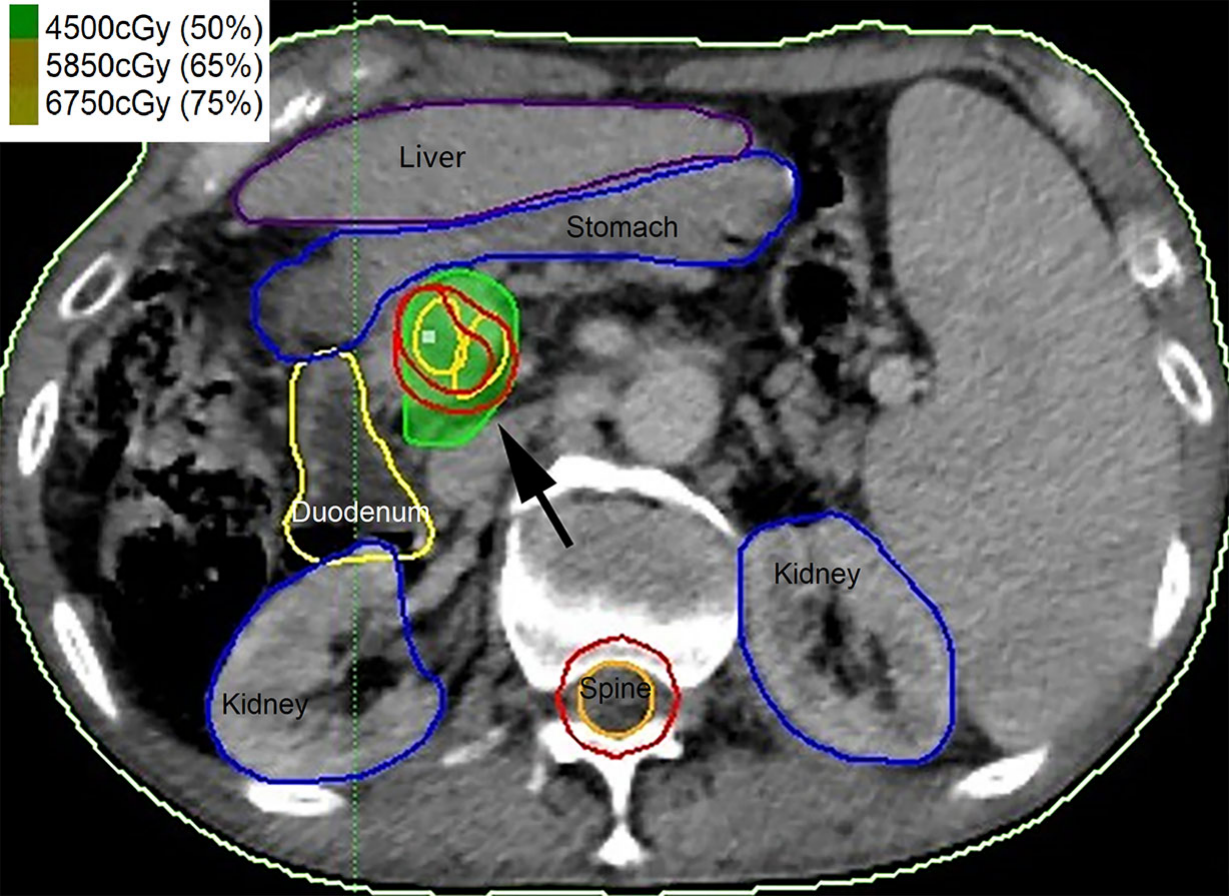
Contouring of the portal vein tumor thrombosis (PVTT) (black arrow) and organ at risk (OAR) and the treatment plans were developed.

Hypofractionated radiotherapy was performed using a Gamma-knife (OUR-QGD/B Version, Shenzhen Aowo Medical New Technology Co., Ltd) loaded with Cobalt^60^. All patients underwent enhanced CT with a slice thickness of 2 mm for detection of the lesion after placing the stereotactic frame. Contouring of the PVTT and organ at risk (OAR) was then conducted. The treatment plans were developed using treatment plan systems (Shenzhen Aowo Medical New Technology Co., Ltd) (**Figure 1**). The prescription dose was individualized and usually 30–45 Gy delivered in 10–15 fractions. The actual dosimetry parameter of the OARs for the patient is listed in **Table 2**.

**Table 2:** The actual dosimetry parameter of the OARs for the 16 patients.

Patients	Stomach Dmax (cGy)	Duodenum Dmax (cGy)	Liver mean dose (cGy)	Cord Dmax (cGy)
1	3,604.4	2,809.4	1,445.2	507.6
2	2,709.1	3,200.6	1,167.2	669.1
3	3,098.2	2,760.6	1,203.4	612.9
4	2,908.5	3,400.7	1,156.7	709.8
5	2,557.8	2,908.7	1,321.5	700
6	3,120.6	3,320.5	899.2	601.6
7	1,600.8	3,609.5	1,006.7	907.6
8	2,099.5	2,500.8	954.5	812.9
9	1,870.5	3,509	1,207.7	615
10	2,234.9	3,621.3	1,165.5	652
11	1,866.3	3,456.7	1,098.4	602.6
12	1,504.7	2,318.9	1,149.6	737.9
13	3,550.8	2,710.6	1,151.4	281.5
14	1,455.6	2,730.5	1,045.7	723.6
15	3,145.4	3,579.9	987.7	908.8
16	3,500.6	3,764.8	1,380.4	642.8

Dmax, maximum dose; OARs, organs at risk.

### Follow-Up

The patients were routinely followed within the first month, 2- to 3-month intervals within the first 2 years, and 3- to 6-month intervals thereafter. Enhanced CT/MRI was conducted to assess the response of the tumor 1–3 months after hypofractionated radiotherapy.

### Statistical Analysis

The data were expressed as mean ± standard deviation or median (interquartile range, IQR). The TTP and overall survival analysis were evaluated by the Kaplan–Meier method. SPSS statistical software (version 26.0) was used.

## Results

### PVTT Response

**Figure 2:**
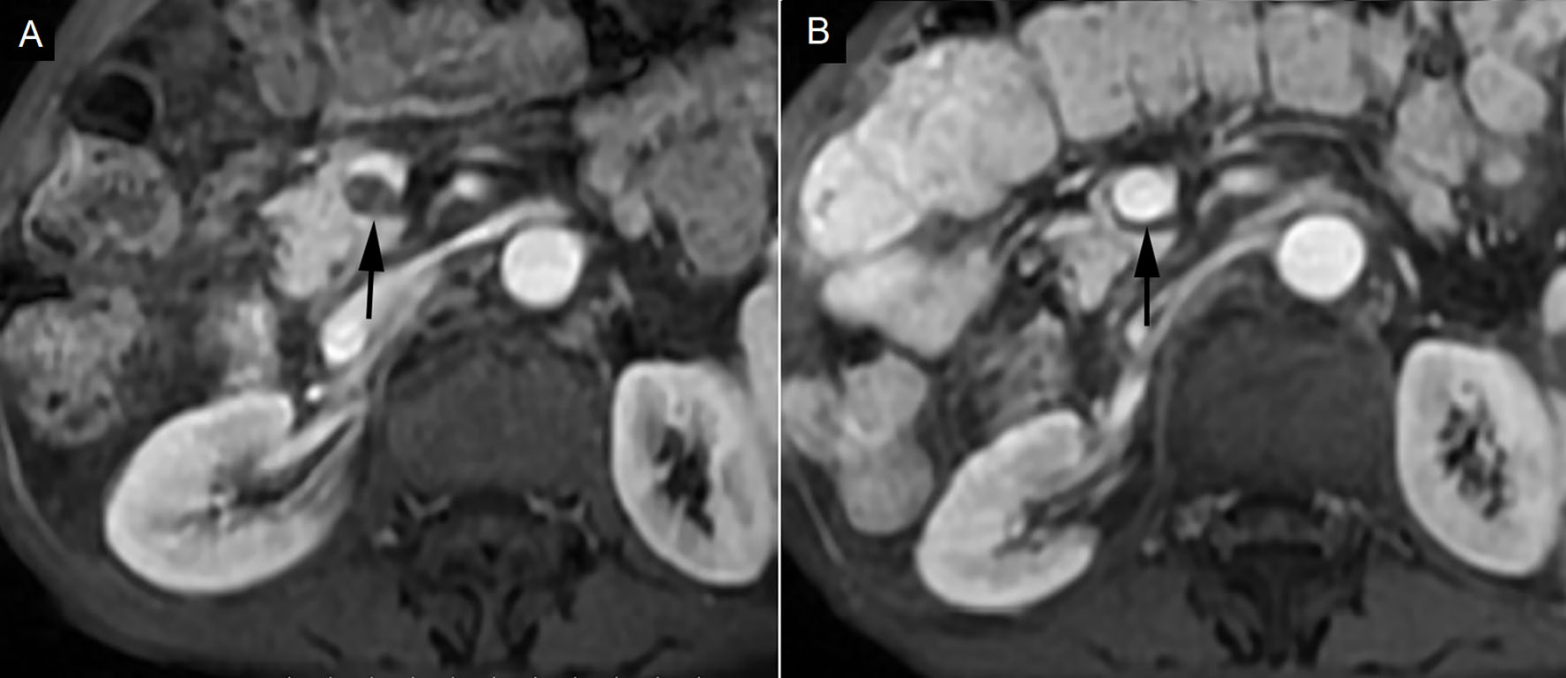
Complete response in a patient with portal vein tumor thrombosis (PVTT). **(A)** PVTT was shown in the main portal vein (black arrow). **(B)** PVTT was eliminated 3 months after hypofractionated radiotherapy (black arrow).

Complete response (CR) was observed in 4 cases (25%), partial response (PR) was observed in 7 cases (43.75%), stable disease (SD) was observed in 5 cases (31.25%), and progressive disease (PD) was not found (**Figure 2**). Symptom relief was observed in all 7 patients with ventosity.

### Treatment Failure and Overall Survival

The median TTP was 6 months (IQR: 6–12 months). Eight patients (50%) failed due to primary cancer progression, 7 patients failed due to extrahepatic metastasis, and only 1 patient failed due to PVTT progression (patient no. 12).

**Figure 3:**
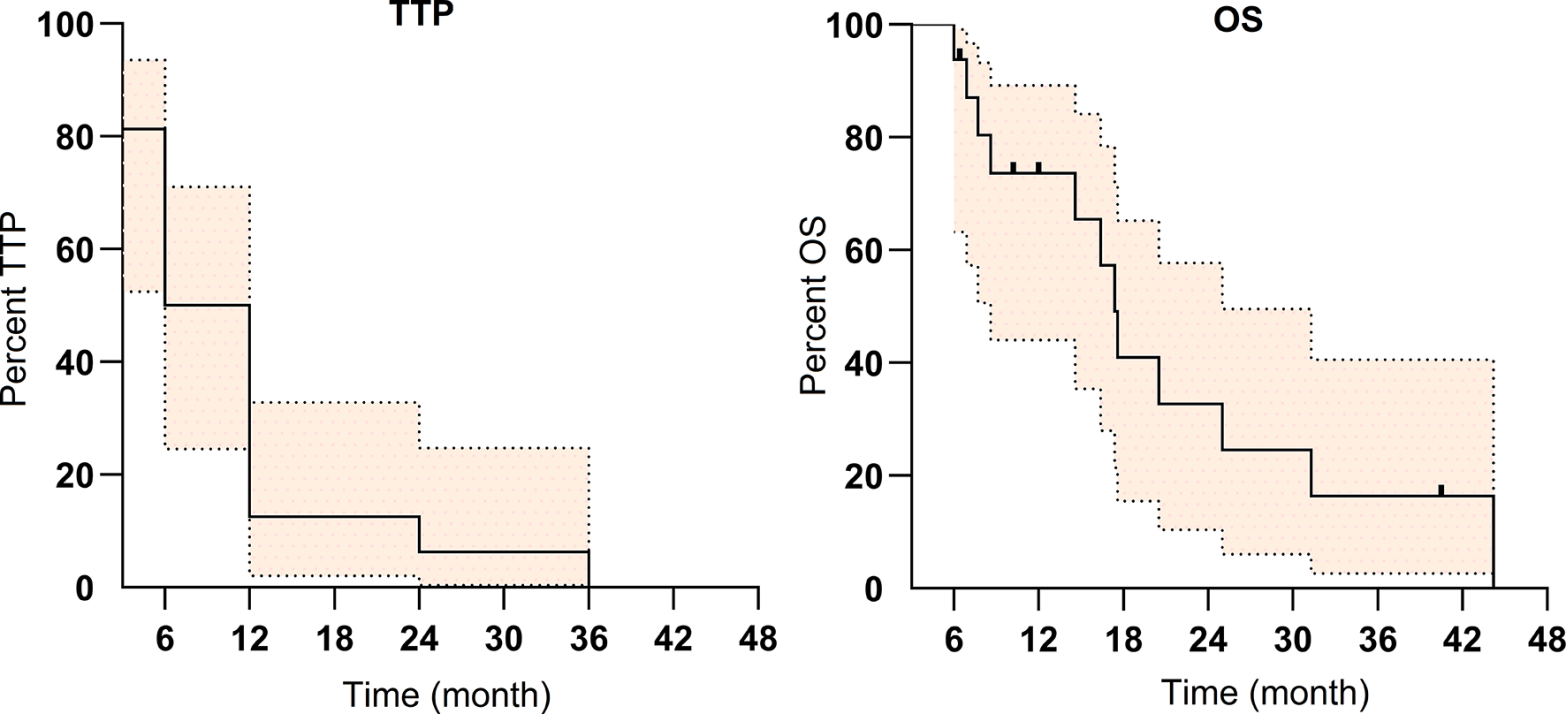
The Kaplan–Meier survival curve of time to progression (TTP) and overall survival (OS) for the 16 patients with portal vein tumor thrombosis (PVTT) (colored area refers to 95% confidence interval).

The median overall survival was 17.4 months (IQR: 8–25 months) (**Figure 3**). Patients with CR/PR were associated with longer OS (6–36 months) compared with that of SD (3–6 months).

Until January 17, 2021, 4 patients (25%) were still alive and 8 patients died due to cachexia, liver failure, primary tumor progression, extrahepatic metastasis, hepatorenal syndrome, or variceal bleeding.

### Complications

Grade I/II anorexia/nausea was observed in 14 patients (87.5%) and Grade I/II leukopenia was observed in 14 patients (87.5%). All recovered without a prolonged hospital stay. No complications ≥ Grade III were observed (**Table 1**).

## Discussion

In the retrospective study of 16 patients, hypofractionated radiotherapy as palliative treatment appears effective and safe for PVTT located in the main portal vein only in cirrhotic patients with advanced hepatocellular carcinoma yielding a high rate of tumor response.

Both the response rate of PVTT and patient survival in the present study were superior to the previously published studies involving EBRT for PVTT (14). In an overview reported by Lee et al. (14), the response rate of three-dimensional conformal radiotherapy (3D-CRT) delivered to PVTT alone was about 50%–75% and that was 40%–50% when delivered to both the PVTT and primary cancer (14). The median survival was reported at 7–8 months for EBRT delivered to PVTT alone and 5 months for non-responders/20 months for responders when delivered to both the PVTT and primary cancer (14). Kim et al. reported hypofractionated radiotherapy using helical tomotherapy for PVTT in 35 patients, and there was a CR in 5 patients (14.3%), PR only in 10 patients (28.6%), SD in 18 patients (51.4%), and PD in 2 patients (5.7%) (16). These historical data were all inferior to that of the present study. The reason for the difference may be that the PVTT located in the main portal may benefit more from focused radiotherapy (23, 24). In the present study, the combination with other treatments, e.g., transarterial chemoembolization in some patients (25–27), and relative high response rate of PVTT may contribute to the relatively long survival time of the patients (median 17.4 months), as responders seem to have significantly lived longer than non-responders (22.0 months vs. 5.0 months) (28, 29). Treatment-related toxicities were reported in most studies, but the specific toxic effect of PVTT treatment was seldom found, and most cases were of non-specific liver toxicities or were associated with RT-related toxicity (14). The complications in the present study were also non-specific and all recovered without a prolonged hospital stay.

Although hypofractionated EBRT or stereotactic body radiotherapy (SBRT) for primary HCC targeting has been broadly studied, the use of these regimens for PVTT treatment has rarely been reported (14). In a multi-center analysis by Lou et al. (17), 75 patients with HCC and inferior vena cava/right atrium tumor thrombus who underwent hypofractionated radiotherapy were retrospectively reviewed. The tumor thrombus completely disappeared (CR) in 17 patients (22.7%), 55 patients (73.3%) had a PR, and 3 patients (4.0%) had an SD, which seems superior to the current study. Patients with inferior vena cava thrombus treated with EBRT tend to have a better response rate and longer survival than those with PVTT (1). A study by Wu et al. evaluated the efficacy of 3-dimensional conformal hypofractionated radiotherapy combined with transcatheter arterial chemoembolization for PVTT; radiotherapy was performed at an exposure of 4–8 Gy/time, 3 times/week, 48–60 Gy, 8–12 times, 3.0–3.5 weeks. The objective response was 71.4%. The overall survival rates were 59.3%, 31.6%, and 26.6% at 1, 2, and 3 years, respectively, with a median survival time of 11 months, which were similar to that reported here and were superior to the previously published studies involving conventionally fractionated radiotherapy for PVTT. Further study is warranted for hypofractionated radiotherapy to evaluate the potential benefit over conventionally fractionated radiotherapy.

PVTT is one of the treatment dilemmas that need to be addressed for patients with HCC. With the advance in modern radiotherapies, such as MRI-guided radiotherapy, helical tomotherapy, or charged particle hypofractionated radiotherapy, focused radiotherapy with precise dose carving and the high prescription dose may be delivered and may shed light on the fields (16, 30, 31). As indicated by the current studies, focused radiotherapy may benefit the patients with high local control. However, primary cancer progression, extrahepatic metastasis, poor liver function, and related events could still be stumbling blocks on patients’ survival improvement. In particular, for patients with HCC and Child–Pugh class B, owing to borderline liver function, any intervention might be offset by liver function deterioration (32), and these patients also yield relatively poor TTP in the present study. Though hypofractionated radiotherapy shows acceptable toxicity in the present study, careful attention should be paid to low-dose volumes that could potentially result in increased liver toxicity (33). Therefore, stricter patient selection may maximize the potential benefits of this treatment (16). In the phase III RESORCE trial, median OS from the start of sorafenib to death was 19.2 months and 26.0 months for patients with sequential regorafenib treatment (34). The median OS (17.4 months) in the present study seems to approach that of sorafenib and significantly inferior to patients with sequential regorafenib treatment. However, only two patients in the present study received systemic therapies (Lenvatinib and anti-PD-1) (35). Therefore, the combination of hypofractionated radiotherapy with a moderate systemic therapy, e.g., immune checkpoint blockade, is warranted (7).

There are several limitations of this study. Firstly, the study is a retrospective study with a small group of patients, which may lead to a certain bias. Secondly, the hypofractionated radiotherapy regimen varied for the patients and lacked a control group with conventionally fractionated radiotherapy. Therefore, the potential benefit of hypofractionated radiotherapy using Gamma-knife over conventionally fractionated radiotherapy was impossible to verify while a relatively high response rate was observed. Finally, the PVTT diagnosis was based on an enhanced CT and MRI image without pathological confirmation and may be biased by the potential onset of portal vein thrombosis. However, this is currently the accepted diagnostic regimen.

## Conclusion

Hypofractionated radiotherapy as palliative treatment appears effective and safe for PVTT located in the main portal vein in cirrhotic patients with advanced hepatocellular carcinoma, yielding a high rate of tumor response. Further study is warranted.

## Data Availability Statement

The raw data supporting the conclusions of this article will be made available by the authors, without undue reservation.

## Ethics Statement

The studies involving human participants were reviewed and approved by Chifeng Tumor Hospital institutional review board. The ethics committee waived the requirement of written informed consent for participation.

## Author Contributions

FF and PZ are responsible for the data collection. FF and BQ are responsible for data analysis and drafting the manuscript. JW and PZ are responsible for reviewing and revising the manuscript and supervising the project. All authors contributed to the article and approved the submitted version.

## Funding

The National Key Research and Development Program of China (Grant No. 2019YFB1311300 to JW) supports the implementation (e.g., labor cost and data collection) and publication of the project.

## Conflict of Interest

The authors declare that the research was conducted in the absence of any commercial or financial relationships that could be construed as a potential conflict of interest.

## Publisher’s Note

All claims expressed in this article are solely those of the authors and do not necessarily represent those of their affiliated organizations, or those of the publisher, the editors and the reviewers. Any product that may be evaluated in this article, or claim that may be made by its manufacturer, is not guaranteed or endorsed by the publisher.

## Abbreviations

PVTT, portal vein tumor thrombosis; SD, stable disease; IQR, interquartile range; HCC, hepatocellular carcinoma; EBRT, external beam radiation therapy; TACE, transarterial chemoembolization; ECOG, Eastern Cooperative Oncology Group; TTP, time to progression; CT, computed tomography; MRI, magnetic resonance imaging; CTCAE, Common Terminology Criteria for Adverse Events; BCLC, Barcelona Clinical Liver Cancer; ESMO, European Society for Medical Oncology; OAR, organ at risk; CR, complete response; PR, partial response; SD, stable disease; PD, progressive disease; 3D-CRT, three-dimensional conformal radiotherapy.
